# Socioeconomic inequalities in health behaviors in children and adolescents: evidence from an Australian cohort

**DOI:** 10.1186/s12889-025-21472-6

**Published:** 2025-01-24

**Authors:** Nirmal Gautam, Mohammad Mafizur Rahman, Rasheda Khanam

**Affiliations:** 1 School of Business, University of Southern Queensland, Toowoomba, QLD 4350 Australia; 2 The Centre for Health Research, University of Southern, Queensland, Toowoomba, QLD 4350 Australia; 3 School of Business, Centre for Health Research, University of Southern Queensland, Toowoomba, QLD 4350 Australia

**Keywords:** Socioeconomic, Inequalities, Health behaviors, Relative index inequality

## Abstract

**Background:**

Understanding the association between socioeconomic inequalities and health behaviors is imperative for elucidating and effectively addressing health inequities among children and adolescents. Despite the wealth of literature on social gradients in health behaviors, longitudinal analyses of socioeconomic inequalities in the health behaviors of children and adolescents are relatively limited, particularly in the Australian literature. Therefore, this study aimed to investigate the association between socioeconomic inequalities and health behaviors among Australian children and adolescents.

**Methods:**

This study utilized the secondary data from the Longitudinal Study of Australian Children (Waves 2–8), which included participants aged 2 to 15 years. Relative index inequality (RII) methods were used to investigate the associations between socioeconomic inequalities and the health behaviors of children and adolescents.

**Results:**

Compared with their counterparts, children and adolescents with high socioeconomic status (SES) are 84% more likely to consume fruits and vegetables (RII = 1.84, 95% CI = 1.63–2.09) and 19% less likely to consume sugary beverages (RII = 0.81, 95% CI = 0.77–0.86), but more likely to consume sweet and savory foods (RII = 1.09, 95% CI = 1.01–1.19). Children and adolescents with high SES were less likely to spend their free time on screens (RII = 0.86, 95% CI = 0.81–0.91) and more inclined toward outdoor activities (RII = 1.75, 95% CI = 1.53–1.98).

**Conclusion:**

This study provides useful insight into socioeconomic inequalities and health behavior outcomes in children and adolescents. These findings stress the need for tailored interventions designed to improve the health behaviors of families from lower socioeconomic backgrounds. Additionally, addressing unhealthy dietary behaviors, such as the higher consumption of sweet and savory foods among children from higher SES backgrounds, is equally critical. Such comprehensive interventions have the potential to reduce socioeconomic disparities in health behaviors and improve the well-being of the broader population.

**Supplementary Information:**

The online version contains supplementary material available at 10.1186/s12889-025-21472-6.

## Introduction

Health behavior is a composite set of lifestyles that arises from the choices made by individuals, shaped by opportunities in accordance with their socioeconomic status (SES), measured by education, income, and occupation [1]. Individuals with a higher SES have greater access to and are more likely to engage in behaviors that promote health [2]. This includes leveraging healthcare services, regularly participating in preventive screenings and vaccinations, and seeking early diagnosis and treatment, which are directly linked to positive health outcomes [3, 4]. On the other hand, those from lower SES backgrounds might find themselves more prone to limited access to these preventive services, further widening socioeconomic disparities in overall health outcomes [5].

Additionally, Gautam et al. (2023) indicated that children from lower SES backgrounds are at an elevated risk for adopting negative health behaviors, such as early initiation of smoking, high-energy-dense food consumption, low physical activity, and involvement in drug abuse. Conversely, children and adolescents from higher socioeconomic backgrounds exhibit a higher prevalence of healthy behaviors, such as increased consumption of fruits and vegetables, dairy products, regular breakfast, adherence to a nutritious diet, and engagement in an active lifestyle [2]. Therefore, socioeconomic inequalities shape health behaviors in children and adolescents, which can contribute to chronic conditions such as obesity and cardiovascular disease [6].

Previously, several studies have explored and examined the relationship between SES and health behaviors in the Australian population [7, 8]. However, longitudinal investigations into how socioeconomic inequalities influence various health behavior outcomes in children and adolescents remain limited [9, 10, 11]. In particular, the Relative Index of Inequality (RII), a crucial measure for quantifying the degree of inequality across different socio-economic strata, offers a more nuanced and comprehensive understanding of disparities in health and suboptimal health behavior outcomes [12, 13, 14]. Furthermore, considering several indicators such as income, education, and employment status offer significant advantages over individual variables. These indicators encapsulate a range of socio-economic conditions that influence health and health behavior outcomes [15]. For instance, income not only affects access to healthcare services but also determines living conditions and nutritional quality, which are critical determinants of health. Education, on the other hand, impacts health literacy and the ability to navigate the healthcare system effectively [16]. By using these indicators, researchers can capture the multi-faceted nature of socio-economic status and its impact on health, leading to more robust and actionable findings [16, 17]. Hence, emphasizing the use of RII and socio-economic indicators allows for a deeper and more accurate analysis of health inequalities, ultimately contributing to the development of targeted interventions and policies [18, 19].

This study will fill gaps in the literature by performing the longitudinal analysis of the socioeconomic inequalities in the health behavior inequalities of Australian children and adolescents. Longitudinal studies provide a nuanced understanding of how health behaviors evolve over time in response to changing socioeconomic conditions, capturing both immediate and delayed effects of inequalities. Such analyses can reveal critical intervention points, particularly during childhood and adolescence when behaviors are most malleable. This study analyzed health behaviors in children and adolescents by categorizing them into healthy and unhealthy behaviors, and offers a nuanced perspective, delineating between actions that promote health and those that pose risks. Such insights are pivotal for policymakers and healthcare professionals in designing targeted intervention strategies aimed at addressing the unique needs of various socioeconomic groups and reducing health inequalities.

## Methods and materials

This study employed data from the birth cohort of the Longitudinal Study of Australian Children (LSAC). The LSAC is an ongoing nationally representative survey, commenced in 2004 and is conducted by the Australian Bureau of Statistics (ABS), the Australian Institute of Family Studies (AIFS), and the Department of Social Service (DSS). The LSAC employed a cross-sequential study design that utilized a multistage cluster sampling method and collected the data biennially, primarily from the biological mother, who serves as the primary caregiver in 95% of cases. If the biological mother was unavailable, data were collected from fathers, grandparents, adoptive parents, and stepparents. Adolescents aged 12 and older provided their data directly to the LSAC. The data were collected using a structured questionnaires for both parents and adolescents [20].

In this secondary data analysis study, we examined data on parental SES and children’s health behaviors collected from primary caregivers when the children were aged 2 to 11 years during waves 2 to 6, and from adolescents aged 12–13 and 14–15 years during waves 7 and 8, respectively. During the 12 years of follow-up, six data points were considered. The initial dataset at wave 2 included 4,605 participants during the years 2005/06. Follow-ups continued through wave 8, culminating in a final sample of 3,127 participants in the years 2017/18. Further details on the LSAC methodology can be found in elsewhere [21].

### Dependent variables

The dependent variable of this study was health behavior, either through healthy behavior or unhealthy behaviors. Healthy behaviors refers to consuming fruits and vegetables and engaging in outdoor and physical activity during leisure time [22]. Consuming sweet and savory foods, sugary drinks, smoking tobacco, drinking alcohol, experiencing sleep problems, engaging in sedentary activities during leisure time, are considered unhealthy behaviors in children and adolescents [23]. Based on prior research [2], this study used seven variables to describe health behavior among children and adolescents: (i) consumption of fruits and vegetables, (ii) intake of sugary beverages, (iii) consumption of sweet and savory food, (iv) movement behaviors, (v) outdoor activities with relatives/parents, (vi) sleeping problems and (vii) smoking tobacco and drinking alcohol. While consumption of fruits and vegetables, intake of sugary beverages, consumption of sweet and savory foods, movement behaviors, outdoor activities with relatives/parents, and sleeping problems were assessed at ages 2 to 15 years (i.e., Waves 2 to 8), smoking tobacco and drinking alcohol were only assessed in adolescents aged 12–13 and 14–15 years during Waves 7 and 8.

### Consumption of fruits and vegetables, sugary beverages, and sweet and savory foods

In the LSAC, the consumption of fruits and vegetables was measured by the following question: (i) *within 24 h*, *how many times did the study child eat cooked vegetables*, *raw vegetables*, *or fresh fruits*? Whereas consumption of sweet and savory foods was measured by (ii) *within 24 h*, *how many times does the study child eat foods including meat pies*, *hamburgers*, *hotdogs*, *sausages*, *sausage rolls*, *French fries*, *savory snacks*, *biscuits*, *doughnuts*, *and chocolates*? Similarly, the consumption of sugary beverages was measured by (iii) *how many times did the study child drink sweet fruit juices*, *Coca-Cola*, *cordial*, *or lemonade within 24 h?* All responses were recorded on an ordinal scale: (i) not at all, (ii) once in the last 24 h, and (iii) twice or more than twice in the last 24 h. Afterward, we converted these responses into binary scales: (1) “not at all” consumption of fruits/vegetables, sweet/savory foods, and sugary drinks was coded as “0”; (2) consumption of fruit/vegetable, sweet/savory food, and sugary foods one or more times a day was coded as “1”.

### Movement behaviors

The LSAC gathered data regarding the movement behaviorsof the study participants, which revealed that the children engaged in various recreational activities during the leisure time, such as riding bikes, dancing, walking, watching TV, painting, drawing, playing video games, and using electronic devices. All responses were recorded as follows: (i) “*Usually*, *chooses inactive pastimes such as TV*, *computer*, *drawing or reading”*, (ii) “*Just as likely to choose active as inactive pastimes”*, and (iii) “*Usually*, *chooses active pastimes such as bike riding*, *dancing*, *games or sports”.* Subsequently, movement behaviors were classified based on physical engagement during free time as biking, dancing, or walking (coded as 1) and screen time and inactive pastimes (including TV watching or electronic device use) (coded as 0).

### Outdoor activities with relatives/parents

The LSAC measured the outdoor activities of children and adolescents by evaluating five distinct types of activities. These included (i) “*watching sports events with parents and other family members”*, (ii) “*going swimming with parents and other family members”*, (iii) “*attending school or community events with parents and other family members”*, (iv) “*visiting the library with parents and other family members”*, and (v) “*attending religious services with parents and other family members”*. All the responses were recorded using a four-point scale, with “No outdoor activities at all” coded as 0, “One or two times in a month” coded as 1, “Two to three times in a month” coded as 2, and “More than four times in a month” coded as 3. These activities were categorized into binary scales: no outdoor activities at all (coded as 0) and one or more than one time in a month (coded as 1).

### Sleeping problems

In the LSAC, sleeping problems of children and adolescents were measured using the *following question: “How much is the study child’s sleeping problem*, *such as not going to bed in time*, *sleeping along*, *walking during night*, *restless sleep*, *bed wetting*, *nightmares*, *and snoring in a* week?”. The responses were “No problem at all” (coded as 0), “One time in a week” (coded as 1), “Two to three times a week” (coded as 2), and “More than three times a week” (coded as 3). Subsequently, we further classified these responses into dichotomous codes. No sleeping problems were coded as 0, and sleeping problems “One time in a week”, “Two to three times a week”, and “More than three times a week” were coded as 1.

### Smoking tobacco and alcohol

The LSAC collected data on risky health behaviors, including smoking tobacco habits and drinking habits, among adolescents aged 12 to 15 years. These behaviors were assessed using the following questions: (i) *“Have you ever smoked even part of a cigarette? “*, (ii) *“Have you ever had an alcoholic drink even?”.* All the responses to the smoking tobacco questionnaire were recorded as (i) “No smoking at all”, coded as 0; “Few puffs fewer than 10 cigarettes in life”, coded as 1; and “More than 100 cigarettes in my life”, coded as 2. Afterward, we further categorized these responses into binary forms: “No smoking at all” was coded as “0”, while “few puffs fewer than 10 cigarettes in life” and “more than 100 cigarettes in my life” were coded as 1.

Furthermore, the adolescents’ alcohol consumption data were collected on four-point Likert scales with “No drinking at all” coded as 0, “Yes, just a few sips” coded as 1, “Yes, I have had fewer than 10 alcoholic drinks in my life” coded as 2, and “Yes, I have had 10 or more alcoholic drinks in my life” coded as 3. Subsequently, we categorized each response into a binary form. The absence of any alcohol consumption was denoted as “0”, while responses indicating minimal alcohol intake (“Yes, just a few sips”), limited alcohol consumption (“Yes, I have had fewer than 10 alcoholic drinks in my life”), and significant alcohol consumption (“Yes, I have had 10 or more alcoholic drinks in my life”) were denoted as “1”.

Detailed information on the health behaviors of the children and adolescents is provided in the Appendix A supplementary file (Table 1).

**Table 1:** Descriptive statistics of study variables

Characteristics	Wave 2 (*n* = 4605)	Wave 3 (*n* = 4386)	Wave 4 (*n* = 4242)	Wave 5 (*n* = 4085)	Wave 6 (*n* = 3764)	Wave 7 (*n* = 3381)	Wave 8 (*n* = 3127)	Missing value(*n*)
**Dependent Variables**	%	%	%	%	%	%	%	%
**Consumption of fruit and Vegetables (e.g.**, **fresh fruits**, **cooked vegetables**, **and raw vegetables) in the last 24 h**								**131(0.6)**
No	108 (3.5)	83 (2.7)	101 (3.2)	126 (4)	281 (9)	287 (9.2)	205 (6.8)	
Yes	3019 (96.5)	3044 (97.3)	3026 (96.8)	3001 (96)	2846 (91)	2840 (90.8)	2791 (93.2)	
**Consumption of sweet and savory foods (e.g.**, **French fries**, **savory food**, **biscuits**, **and pie) in the last 24 h**								**131(0.6)**
No	354 (11.3)	377 (12.1)	276 (8.8)	295 (9.4)	449 (14.4)	480 (15.4)	477 (15.9)	
Yes	2773 (88.7)	2750 (87.9)	2851 (91.2)	2832 (90.6)	2678 (85.6)	2647 (84.6)	2519 (84.1)	
**Drinking sugary beverages (e.g.**, **fruit juice**, **soft drink/cordial)**								**131(0.6)**
No	945 (30.2)	297 (9.5)	1124 (35.9)	1240 (39.7)	1136 (36.3)	1287 (41.2)	1264 (42.2)	
Yes	2182 (69.8)	2830 (90.5)	2003 (64.1)	1887 (60.3)	1991 (63.7)	1840 (58.8)	1732 (57.8)	
**Movement behaviors**								**250 (1.1)**
Screening time	2047 (65.5)	2181 (69.8)	1784 (57.2)	2247 (72.6)	2353 (76.8)	2587 (83.6)	2526 (83.8)	
Physical activity	1080 (34.5)	942 (30.2)	1337 (42.8)	848 (27.4)	709 (23.2)	508 (16.4)	490 (16.2)	
**Outdoor activities**								**194 (0.9)**
No outdoor activities	89 (2.8)	91 (2.9)	84 (2.7)	112 (3.6)	120 (3.9)	210 (6.9)	373 (12)	
One or more than one time in a month	3038 (97.2)	3035 (97.1)	3039 (97.3)	2985 (96.4)	2946 (96.1)	2841 (93.1)	2732 (88)	
**Sleeping problems**								**210 (1)**
No, sleeping problem at all	1861 (59.5)	2222 (71.1)	2255 (72.2)	2263 (73.1)	2215 (72.4)	2227 (73)	2084 (67.2)	
Yes, sleeping problem	1265 (40.5)	904 (28.9)	867 (27.8)	833 (26.9)	843 (27.6)	823 (27)	1017 (32.8)	
Smoking tobacco								**320(5.1)**
No	-	-	-	-	-	3076 (98.4)	2876 (92)	
Yes	-	-	-	-	-	51 (1.6)	251 (8)	
Drinking alcohol								**354(5.7)**
No	-	-	-	-	-	2373 (75.9)	1662 (53.1)	
Yes	-	-	-	-	-	754 (24.1)	1465 (46.9)	
**Key independent variables**								
**Household’s income**								**71(0.3)**
Lowest income	1625 (52)	1346 (43)	1210 (38.7)	1100 (35.3)	1014 (32.5)	960 (31.3)	893 (28.6)	
lowest to medium income	1176 (37.6)	1320 (42.2)	1387 (44.4)	1295 (41.5)	1239 (39.7)	1130 (36.8)	1094 (35)	
Medium to highest income	276 (8.8)	391 (12.5)	454 (14.5)	632 (20.3)	761 (24.4)	831 (27.1)	953 (30.5)	
Highest income	50 (1.6)	70 (2.2)	76 (2.4)	93 (3)	109 (3.5)	150 (4.9)	183 (5.9)	
**Mother education**								**4406(20.1)**
Postgraduation	235 (10.4)	251 (10.6)	265 (10.9)	296 (11.8)	303 (11.6)	331 (12.6)	360 (13.3)	
Undergraduate	826 (36.5)	868 (36.7)	884 (36.5)	902 (36.1)	908 (34.9)	905 (34.4)	939 (34.8)	
Certificate/Diploma	1146 (50.6)	1190 (50.3)	1221 (50.4)	1253 (50.1)	1342 (51.6)	1339 (50.9)	1323 (49.1)	
Year 12 or below	59 (2.6)	55 (2.3)	53 (2.2)	50 (2)	50 (1.9)	54 (2.1)	75 (2.8)	
**Mother employment**								**134 (0.6)**
Employed	1837 (58.7)	2054 (65.7)	2167 (69.5)	2326 (74.7)	2433 (78.2)	2499 (81.9)	2662 (85.7)	
unemployed	92 (2.9)	59 (1.9)	73 (2.3)	94 (3)	93 (3)	84 (2.8)	61 (2)	
Not in labor force	1198 (38.3)	1011 (32.4)	880 (28.2)	694 (22.3)	586 (18.8)	468 (15.3)	384 (12.4)	
***Control Variables***								
**Gender**								**-**
Male	1581 (50.6)	1581 (50.6)	1581 (50.6)	1581 (50.6)	1581 (50.6)	1581 (50.6)	1606 (51.4)	
Female	1546 (49.4)	1546 (49.4)	1546 (49.4)	1546 (49.4)	1546 (49.4)	1546 (49.4)	1521 (48.6)	
**Place of Residence**								**-**
Accessible	2980 (95.3)	2980 (95.3)	2980 (95.3)	2980 (95.3)	2980 (95.3)	2980 (95.3)	3084 (98.6)	
Not accessible	147 (4.7)	147 (4.7)	147 (4.7)	147 (4.7)	147 (4.7)	147 (4.7)	43 (1.4)	
**Ethnicity**								**-**
No Aboriginal and Torres landler	3016 (96.5)	3025 (96.7)	3021 (96.6)	3023 (96.7)	3044 (97.3)	3051 (97.6)	3048 (97.5)	
Aboriginal and Torres landler	111 (3.5)	102 (3.3)	106 (3.4)	104 (3.3)	83 (2.7)	76 (2.4)	79 (2.5)	
**Languages spoken**								**-**
English	2659 (85)	2692 (86.1)	2707 (86.6)	2886 (92.3)	2839 (90.8)	2855 (91.3)	2859 (91.4)	
Other than English	468 (15)	435 (13.9)	420 (13.4)	241 (7.7)	288 (9.2)	272 (8.7)	268 (8.6)	
**Number of Siblings**								**71(0.3)**
One child	2133 (68.2)	1819 (58.2)	1679 (53.7)	1611 (51.6)	1651 (52.9)	1637 (53.3)	1738 (55.7)	
Two children	701 (22.4)	916 (29.3)	995 (31.8)	1027 (32.9)	1001 (32.1)	981 (31.9)	972 (31.1)	
Three children	213 (6.8)	285 (9.1)	339 (10.8)	352 (11.3)	343 (11)	332 (10.8)	314 (10.1)	
four children	80 (2.6)	107 (3.4)	114 (3.6)	130 (4.2)	128 (4.1)	121 (3.9)	99 (3.2)	
**General health status**								**137(0.6)**
Good health	3047 (97.4)	3067 (98.1)	3068 (98.2)	3043 (98.2)	3011 (98.2)	3042 (98)	3012 (97)	
Poor health	80 (2.6)	59 (1.9)	56 (1.8)	55 (1.8)	56 (1.8)	62 (2)	94 (3)	

Note: Lowest income (500 AUD or less per week); lowest to medium (501 to 999 AUD per week); Medium to highest (1000 to 1999 AUD per week); Highest (more than 2000 AUD per week). Active time refers to riding a bike/dancing/ walking, and screening time refers to watching mobile, video, and TV

## Independent variables

### Measure of socioeconomic status

SES is a multidimensional construct used in social, epidemiological, and health economic research and has been assessed since the 19th century [24, 25, 26]. It is defined objectively by factors such as income, education, and occupation of the individuals, and subjectively, it is evaluated based on social status and social class, place of residence, ethnic origin, religious and geographical background [24]. In this study, we assessed SES using three key indicators: income, education, and employment from wave 2 to wave 8 (Appendix A: Table 2). Each indicator was standardized to z scores (mean = 0, SD = 1) to ensure comparability. A composite SES score was then calculated as the mean of these standardized indicators following methods used in similar studies for comprehensive SES assessment [27, 28]. Afterward, we calculated the relative rank ${r_i}\,=\frac{1}{N}$ as the mean of a fractional rank score of the study participants ranked by education, income, and employment (i.e., $i=1$ for deprived individuals, and$i=N$ for affluent individuals). The mean of the fractional rank score serves as the cutoff point for determining an individual’s SES. A higher mean fractional rank score indicates a higher SES, whereas a lower mean score signifies a lower SES. This approach, validated in prior research, accurately reflects individuals’ positions in the socioeconomic spectrum [29, 30, 31]. This relative rank was utilized as the key independent variable (i.e., SES) in regression models to evaluate the association between socioeconomic inequalities and health behaviors.

**Table 2:** Association between socioeconomic inequalities and health behaviors in children and adolescents(*n* = 3127)

Variables	Consumption of Fruit and vegetables (Yes/No)	Consumption of sweet and savory food (Yes/No)	Drinking sugary beverages (Yes/No)	Movement behaviors (Screentime/ Active time	Outdoor activities with relatives/parents (Yes/No)	Sleep problem (Yes/No)	Smoking tobacco (Yes/No)	Drinking alcohol (Yes/No)
	OR (95%CI)	OR (95%CI)	OR (95%CI)	OR (95%CI)	OR (95%CI)	OR (95%CI)	OR (95%CI)	OR (95%CI)
**Relative index inequalities (RII)**	1.84(1.63–2.09)	1.09(1.01–1.19)	0.81(0.77–0.86)	0.86(0.81–0.91)	1.75(1.53–1.98)	0.91(0.85–0.96)	0.85(0.67–1.11)	0.99(0.89–1.11)
**Socioeconomic status (Ref: low SES)**								
High SES	2.03(1.78–2.29)	1.11(1.01–1.19)	0.85 (0.80–0.90)	0.90 (0.84–0.96)	1.81 (1.59–2.07)	0.95(0.89-1)	0.81(0.63–1.02)	0.94 (0.85–1.06)
**Age Groups (Ref: children)**								
Adolescence	0.45(0.22–0.92)	0.67 (0.51–0.88)	0.59 (0.23–1.46)	0.42(0.27–0.66)	0.29 (0.19–0.44)	0.97(0.69–1.35)	-	-
**Gender (Ref: Male)**								
Female	1.08(0.95–1.22)	0.95 (0.88–1.03)	0.91(0.85–0.96)	1.01(0.94–1.07)	1.05(0.92–1.19)	0.97(0.91–1.02)	0.96 (0.76–1.21)	1.08 (0.97–1.21)
**Place of residence (Ref: accessible)**								
Not accessible	0.97(0.72–1.31)	0.91(0.74–1.11)	0.96 (0.83–1.11)	0.91(0.78–1.06)	0.96 (0.69–1.34)	0.93(0.80–1.08)	1.29 (0.61–2.71)	1.01 (0.73–1.41)
**Ethnicity (Ref: no Aboriginal)**								
Aboriginal	0.55(0.42–0.73)	1.02(0.81–1.31)	1.66(1.38–2.01)	1.26 (1.07–1.50)	0.63 (0.47–0.85)	1.31(1.11–1.54)	2.71 (1.62–4.54)	0.89 (0.63–1.26)
**Languages spoken (Ref: English**)								
Other than English	1.51(1.21–1.88)	0.89 (0.78–1.01)	1.24 (1.13–1.37)	0.87 (0.79–0.96)	0.92(0.75–1.12)	0.91(0.82-1)	0.51(0.30–0.87)	0.66(0.54–0.81)
**Number of Siblings (Ref: One child)**								
Two children	1.25(1.08–1.42)	1.08(0.98–1.18)	0.90 (0.84–0.96)	1.09(1.02–1.17)	1.21 (1.04–1.39)	0.91 (0.85–0.97)	0.98(0.75–1.28)	0.91(0.81–1.03)
Three children	1.21(0.98–1.47)	1.11(0.96–1.28)	0.96(0.87–1.06)	1.1(0.99–1.22)	1.03 (0.84–1.27)	0.79(0.71–0.88)	0.93(0.62–1.39)	0.90(0.74–1.08)
four children	1.25 (0.91–1.71)	1.22 (0.96–1.54)	0.81(0.69–0.96)	1.29(1.09–1.51)	0.95 ( 0.70–1.29)	0.76(0.64–0.90)	1.01(0.52–1.91)	0.95(0.70–1.28)
**General health status (Ref: Excellent)**								
Poor health	0.57(0.41–0.79)	0.89(0.68–1.16)	0.85(0.69–1.03)	1.11(0.90–1.36)	0.68 (0.48–0.96)	3.19(2.64–3.86)	2.32 (1.37–3.91)	1.01(0.71–1.41)

Note: OR = odds ratio, CI = confidence interval

### Control variables

These variables included age group, gender (i.e., male or female), place of residence, whether the child identified as Aboriginal or Torres Landler, whether English was spoken at home, the number of children living with parents, the general health of the study child, and the remoteness of the family residence. Details are provided in Table 1.

### Statistical analysis

Initially, this study utilized descriptive statistics, including frequency and mean, to present an overview of the variables under investigation. Second, both the Relative Index of Inequality (RII) and the Generalized Mixed-Effects Regression Model (GLMM) were used to analyze the data. GLMM enables a more comprehensive analysis of longitudinal data. This approach provides a more robust set of statistical tools for longitudinal data analysis, resulting in more powerful hypothesis tests, more accurate estimates of rates of change, and significant advancements in statistical methodologies for analyzing longitudinal data [32, 33]. On the other hand, the RII measured the magnitude of socioeconomic inequalities in health behaviors. The RII is a regression-based measure that quantifies the extent of inequality in health outcomes across the socioeconomic spectrum. It expresses the ratio of health outcomes between individuals at the theoretical lowest and highest positions of SES.

In this study, we used three key socioeconomic indicators: education, income, and employment status. Each individual’s rank for these indicators was determined, creating a fractional rank as the cutoff point for determining their position within the socioeconomic hierarchy. These fractional ranks were used as predictors in a GLMM, which is suitable for binary health outcomes. The RII was derived from the odds ratios (ORs) produced by the GLMM, where the ORs reflect the relative likelihood of experiencing a health outcome based on socioeconomic status. The RII provides a summary measure of the inequality, representing the difference in health outcomes between those at the bottom and top of the SES distribution [12].

In addition to the primary analysis, sensitivity analyses were conducted to test the robustness of the results. Specifically, we evaluated the association of each socioeconomic indicator (income, education level, and employment status) individually with health behavior (See online Appendix B). Additionally, instances of missing data were addressed through simple imputation techniques, with mean imputation for continuous data and mode for categorical data. All the statistical procedures were performed using R software.

## Results

### Descriptive statistics of the study variables

In Wave 2, 96.5% of participants reported consuming fruits and vegetables in the past 24 h, but this figure decreased to 93.2% by Wave 8. There was also a decline in the consumption of sweet and savory foods, from 88.7 to 84.1%, and sugary beverages, from 69.8 to 57.8%, over the same period. Physical activity and outdoor activity participation showed fluctuations, with physical activity peaking at 42.8% in Wave 4 and outdoor activity at 97.2% in Wave 2. However, by Wave 8, participation in physical activity had significantly dropped to 16.2%, and outdoor activity participation had decreased to 88%. Additionally, screen time increased from 65.5 to 83.8% across the waves. Regarding risk behaviors such as smoking tobacco and alcohol consumption, data were available only for the later waves. By Wave 8, 8% of participants reported smoking tobacco, and 46.9% reported alcohol consumption. For more detailed information(Table 1).

Table 1: **here**.

### Associations between socioeconomic inequalities and the consumption of fruits and vegetables, sugary beverages, and sweet and savory foods

The analysis highlighted that socioeconomic inequalities were strongly associated with disparities in dietary habits, particularly in the consumption of fruits, vegetables, and sugary beverage. Individuals from high SES backgrounds were 84% more likely to consume fruits and vegetables than their low SES counterparts (RII = 1.84, 95% CI = 1.63–2.09), and were 19% less likely to consume sugary beverages (RII = 0.81, 95% CI = 0.77–0.86). Furthermore, the results show 9% greater inequality in the consumption of sweet and savory foods among the compared groups, indicating that participants from high SES backgrounds were more likely to consume sweet and savory foods (RII = 1.09, 95% CI = 1.01–1.19) compared with their counterparts (Table 2).

For children and adolescents from high SES backgrounds, the odds of consuming fruits and vegetables were 2.03 times (OR = 2.03, 95% CI 1.78–2.29) higher compared with their counterparts. Conversely, the odds of consuming sugary beverages were 0.85 times (OR = 0.85, 95% CI 0.80–0.90) lower for children and adolescents from high SES backgrounds than their low SES counterparts.

### Associations between socioeconomic inequalities and movement behaviors, outdoor activity, and sleeping problems

Individuals with higher SES were less likely to spend their free time on screens (RII = 0.86, 95% CI = 0.81–0.92) and were significantly more inclined toward outdoor activities with relatives/parents (RII = 1.75, 95% CI = 1.53–1.98). Additionally, those with high SES were less likely to have sleep problems (RII = 0.91, 95% CI = 0.85–0.96) than those with low SES. In relation to the associations between SES and movement behaviors, outdoor activity with relatives/parents, and sleeping problems, children and adolescents from high SES backgrounds had lower odds of spending time on screens (OR = 0.90, 95% CI = 0.84–0.96) compared with their counterparts. However, the data revealed that for children and adolescents with high SES backgrounds, the odds of participating in outdoor activities with relatives/parents were 1.81 times higher (OR = 1.81, 95% CI = 1.59–2.07) than their counterparts. Regarding SES and sleeping problems in children and adolescents, those from high SES backgrounds had lower odds of developing sleeping problems (OR = 0.95, 95% CI = 0.89-1) compared with those from low SES backgrounds (Table 2).

### Associations between socioeconomic inequalities and smoking tobacco and drinking alcohol

In relation to socioeconomic inequalities and smoking tobacco, the RII was 0.85, with a 95% confidence interval ranging from 0.67 to 1.11. This indicates a 15% lower prevalence of smoking tobacco among higher socioeconomic groups compared with lower socioeconomic groups. Alcohol consumption, the RII was 0.99, with a 95% CI from 0.89 to 1.11. However, there was no significant association between SES, smoking tobacco, and drinking alcohol among adolescents (Table 2).

Table 2: **here**.

### Sensitivity analysis

This study performed sensitivity analyses incorporating all SES indicators ( i.e., parental income, education, and employment) with health behavior variables. The result shows that low parental SES significantly influences poor health behaviors in children and adolescents, which is consistent with our main findings. The results of the sensitivity analyses can be found in the online Appendix B.

## Discussion

The results of this study indicate a significant association between socioeconomic inequalities and health behaviors among children and adolescents. Specifically, children and adolescents from lower socioeconomic backgrounds were found to have higher rates of unhealthy behaviors, such as poor dietary habits and lower physical activity levels, compared to their peers from higher socioeconomic backgrounds. These findings highlight the persistent impact of socioeconomic disparities on health behaviors and emphasize the need for targeted public health interventions to address these inequalities.

Aligned with our objective, this study’s findings establish a significant association between high SES and the adoption of healthy behaviors, such as consumption of fruits and vegetables, as well as unhealthy behaviors, including the intake of sweet and savory foods, and sugary beverages among children and adolescents. These observations are consistent with prior research showing that families from high SES backgrounds tend to have healthier diets, but this is not solely due to their financial ability to afford healthier food. According to the literature, several factors contribute to dietary disparity. Higher SES families often have greater access to education, which informs healthier food choices, and they live in neighborhoods with better access to supermarkets that offer a variety of nutritious food options [34]. Additionally, these families might allocate the time and resources to prepare healthy meals, unlike low SES families who may rely on convenience foods due to time constraints and limited access to fresh produce [35, 36].

Furthermore, regarding socioeconomic inequalities and movement behaviors, and outdoor activities with relatives/parents, our study highlights that children and adolescents from high SES backgrounds tend to have less screen time and increased engagement in outdoor activities with relatives/parents. This is possibly due to a combination of factors deeply rooted in their socioeconomic environment. High SES families often have a heightened awareness of the negative effects of excessive screen time, as their educational background provides them with better knowledge about its impact on physical and mental health [37, 38]. Consequently, they actively encourage their children to participate in outdoor activities that promote physical well-being [39]. Additionally, high SES families have the financial resources to access a wide range of outdoor opportunities, from sports clubs to safe recreational areas, which allows their children to participate in structured physical activities [39, 40]. Similar findings were well documented in other literature; children from lower SES families often have limited access to participate in sports, recreational facilities, and community programs, which can reduce their opportunities for outdoor activities, therefore, children from low SES are more inclined to be involved in screen time than their counterparts are [41, 42]. Financial difficulties further exacerbate these disparities, as the costs associated with participation, event attendance, and even transport to these venues could be difficult for families with low SES backgrounds [40]. Moreover, the educational gap also plays a crucial role: lower SES families might lack awareness of the benefits of such activities or how to access available resources and programs [43]. Time constraints are another critical factor, with parents in lower SES households more likely to work multiple jobs or irregular hours, reducing their ability to support their children’s participation in extracurricular activities [44].

By examining socioeconomic inequalities and sleeping problems, our study revealed a negative association between low SES and sleep quality in children and adolescents, indicating that children and adolescents from lower socioeconomic backgrounds tend to have poorer sleep quality. The economic instability and environmental stressors prevalent in lower SES households contribute to sleep disruptions [45, 46]. On the other hand, the literature has reported that families with lower parental education levels frequently encounter economic hardships that contribute to stress-inducing living conditions, directly affecting children’s ability to achieve restful sleep [47, 48]. These conditions include overcrowded housing, exposure to neighborhood noise, and elevated household tension, all of which disturb people’s sleep [49].

However, in this study, we found no significant association between SES, smoking tobacco, and drinking alcohol. This finding contrasts with some prior research that has reported a significant association between SES, smoking tobacco, and drinking alcohol in adolescents. For instance, studies show that individuals with a high SES were less likely to smoke tobacco compared with their counterparts. Parents in high SES families are generally more educated and aware of the health risks associated with smoking, leading to stronger communication about its dangers. These families often promote health-conscious environments with better access to recreational activities. Additionally, higher parental monitoring, effective public health campaigns, and the perception of smoking as wasteful contribute to reducing smoking behaviors among adolescents in these groups [50, 51, 52]. The extant literature supports these findings, highlighting a pronounced predisposition toward smoking tobacco within lower SES populations [53, 54, 55].

On the other hand, Thor et al. (2019) found that individuals with higher SES had a higher chance of drinking alcohol compared to their counterparts. This is possibly due to greater disposable income and social opportunities [56]. Similarly, Kuppens et al. (2020) also found a positive association, attributing higher alcohol consumption among higher SES groups to similar factors [57]. The discrepancy between our findings and prior studies may be due to several factors. Firstly, differences in sample populations, methodological measurements, and social, cultural, and contextual variations could influence the relationship between SES smoking tobacco and alcohol-drinking behavior [58, 59, 60]. Further research is needed to explore these discrepancies and identify potential moderators of the SES-smoking tobacco and alcohol consumption relationship. Understanding these nuances is crucial for developing targeted public health interventions aimed at reducing alcohol-related harm across different socioeconomic groups.

Overall, examining the association between socioeconomic inequalities and the health behaviors of children and adolescents using the LSAC dataset holds significant importance. This study provides a longitudinal analysis of socioeconomic inequalities and health behaviors by analyzing both the healthy and unhealthy spectrum of health behaviors in children and adolescents. The findings from this study suggest potential pathways for targeted interventions and policy adjustments to address inequalities in health behaviors. For instance, public health programs could be designed to specifically target children and adolescents from lower socioeconomic backgrounds, providing them with resources and support to engage in healthier behaviors. Schools and community centers could implement educational programs and activities that promote physical activity and healthy eating, particularly in underserved areas. Additionally, policymakers could use the findings to advocate for a more equitable distribution of resources and access to healthcare services, ensuring that all children and adolescents have the opportunity to achieve optimal health [61, 62, 63]. By addressing the root causes of socioeconomic inequalities, these interventions can contribute to reducing health disparities and improving the overall health and well-being of future generations [64].

### Strengths and limitations

To our knowledge, this study is the first to use 12 years of longitudinal data to investigate the dynamic relationship between socioeconomic inequalities and a wide range of health behaviors in young Australians. The strength of this study is further enhanced by the use of established and recognized instruments for measuring outcome variables. LSAC strictly complies with the leading global standards for longitudinal cohort studies to reduce biases stemming from geographic differences and nonresponses [20, 65]. In particular, the assessment of children’s and adolescents’ health behaviors covered various dimensions, including dietary habits, movement behaviors, outdoor activity with relatives/parents, sleep problems, smoking tobacco and drinking alcohol. However, this study is not without its limitations. The risk of social desirability bias on various aspects of food habits, drinking habits, and physical activity, which may obscure the outcomes. Finally, the limited representation of Aboriginal Australians in the study restricts the generalizability of the findings and might not adequately reflect the unique experiences or conditions of these communities.

## Conclusion

This study underscores the association between socioeconomic inequities and health behaviors in Australian children and adolescents. Leveraging 12 years of data from the LSAC, the study highlights substantial disparities in health behaviors based on SES. High SES is significantly associated with healthier behaviors in children and adolescents, such as increased consumption of fruits and vegetables, reduced intake of sugary drinks, more outdoor activities with relatives/parents, lower screen time during leisure time, and fewer sleep problems. However, unhealthy behaviors, such as increased consumption of sweet and savory foods, were more common among children and adolescents with high SES. To address these disparities, interventions could be considered to enhance access to health education, affordable nutritious food, and recreational opportunities, particularly in low SES communities. By improving the socioeconomic conditions of disadvantaged families and fostering environments that support healthier lifestyles, policymakers can make strides in reducing these health inequalities. This will not only improve health outcomes for children and adolescents but also contribute to long-term public health benefits across the population.

## Electronic supplementary material

Below is the link to the electronic supplementary material.

Supplementary Material 1

## Data Availability

The data used are confidential. Interested parties must comply with certain restrictions and sign confidentiality agreements. To request access to the data, individuals should reach out to the Australian Department of Social Services via the following link: https://dataverse.ada.edu.au/dataverseuser.xhtml?editMode=CREATE.

## Abbreviations

CI: Concentration index
LSAC: Longitudinal Study of Australian Children
OECD: Organization for Economic Co-operation and Development
RII: Relative inequality index
SES: Socioeconomic Status
