# Supplementary material for: Socioeconomic inequalities in health behaviors in children and adolescents: evidence from an Australian cohort
Source: BMC Public Health. 2025 Jan 24;25:314. doi: 10.1186/s12889-025-21472-6 (PMC11762062; doi:10.1186/s12889-025-21472-6)
Supplement: Supplementary file 1 — Supplementary Material 1 [file 12889_2025_21472_MOESM1_ESM.docx]

**Appendix A**

**Table 1: Food habit:** In LSAC, Consumption of fruit and vegetables, fatty food, and the consumption of sugary beverages were measured by following questions and these questions are available in all waves from Wave 2 to Wave 8

| **Questions** | **Response** |
| --- | --- |
| **Consumption of fruit and vegetables** | |
| 1. In the last 24 hours how often did the study child eat fresh fruit? | 0= Not at all  1= Once in the last 24  2= Twice in the last 24  3= 3 or more times in the last 24 |
| 1. In the last 24 hours how often did the study child eat cooked vegetables? | 0= Not at all  1= Once in the last 24  2= Twice in the last 24  3= 3 or more times in the last 24 |
| 1. In the last 24 hours how often did the study child eat raw vegetables or salad? | 0= Not at all  1= Once in the last 24  2= Twice in the last 24  3= 3 or more times in the last 24 |
| **Consumption of sweet and savory foods** | |
| 1. In the last 24 hours has child had the following foods and drinks once, more than once, or not at all? Meat pie, hamburger, hot dog, sausage or sausage roll? | 0= Not at all  1= Once in the last 24  2= More than once in the last 24 |
| 1. In the last 24 hours has child had the following foods and drinks once, more than once, or not at all? Hot chips or French fries? | 0= Not at all; 1= Once; 2= More than once |
| 1. In the last 24 hours has child had the following foods and drinks once, more than once, or not at all? Potato chips or savoury snacks such as 'Twisties' etc? | 0= Not at all; 1= Once; 2= More than once |
| 1. In the last 24 hours has child had the following foods and drinks once, more than once, or not at all? Biscuits, doughnuts, cake, pie or chocolate? | 0= Not at all; 1= Once; 2= More than once |
| **Drinking sugary beverages** | |
| 1. In the last 24 hours has child had the following foods and drinks once, more than once, or not at all? Fruit juice? | 0= Not at all  1= Once in the last 24  2= More than once in the last 24 |
| 1. In the last 24 hours has child had the following foods and drinks once, more than once, or not at all? Soft drink or cordial, not diet? | 0= Not at all  1= Once in the last 24  2= More than once in the last 24 |

**Activities:** In LSAC, free time activities, outdoor activities, and sleeping problems were measured by following questions and these questions are available in all waves from Wave 2 to Wave 8.

| **Questions** | **Response** |
| --- | --- |
| **Free time activities** | |
| 1. What does the study child usually do when he/she has a choice about how to spend free time? | 1= Usually chooses inactive pastimes like TV, computer, drawing or reading 2= Just as likely to choose active as inactive pastimes  3= Usually chooses active pastimes like bike riding, dancing, games or sports |
| **Outdoor activities** | |
| 1. In the past month has the study child done any of these things with you or another family member? Gone to a playground or a swimming pool? | **0= No**  **1= Yes** |
| 1. In the past month has the study child done any of these things with you or another family member? Gone to a concert, play, museum, art gallery or community or school event? | **0= No**  **1= Yes** |
| 1. In the past month has the study child done any of these things with you or another family member? Gone to the cinema or to watch a sporting event? | **0= No**  **1= Yes** |
| 1. In the past month has the study child done any of these things with you or another family member? Attended a religious service, church, temple, synagogue or mosque? | **0= No**  **1= Yes** |
| 1. In the past month has the study child done any of these things with you or another family member? Visited a library? | **0= No**  **1= Yes** |
| **Sleeping problem** | |
| 1. How much is the study child's sleeping pattern or habits a problem for you? | 1= Not a problem at all  2= A small problem  3= A moderate problem  4= A large problem |

**Smoking and alcohol:** In LSAC, leisure time activities, outdoor activities, and sleeping problems were measured by following questions and these questions are available in all waves from Wave 7 and Wave 8.

| **Questions** | **Response** |
| --- | --- |
| **Smoking Tobacco** | |
| 1. Have you ever smoked even part of a cigarette? | 1= No  2= Yes, just a few puffs  3= Yes, I have smoked fewer than 10 cigarettes in my life  4= Yes, I have smoked 10 to 100 cigarettes in my life  5= Yes, I have smoked more than 100 cigarettes in my life |
| **Alcohol** | |
| 1. Have you ever had even part of an alcoholic drink? | 1= No  2= Yes, just a few sips  3= Yes, I have had fewer than 10 alcoholic drinks in my life  4= Yes, I have had 10 or more alcoholic drinks in my life |

**Table 2: Measure of parental socioeconomic status**

| **Topic** | **construct** | **Measure** | **Question** | **Value** |
| --- | --- | --- | --- | --- |
| Finance | Parental Income | Usual income | Weekly or annual income from all sources | 1= Less than AUD500 pw (AUD25,999 or less per year); 2= AUD500-AUD999 pw (AUD26,000-AUD51,999 per year);  3= AUD1,000-AUD1,999 pw (AUD52,000-AUD103,999 per year);  4= AUD2,000 or more per week (AUD104,000 or more per year) |
| Family demographics | Parental education | Completed post-secondary qualification | What is the level of the highest qualification that your partner has ever completed? | 1= Postgraduate degree;  2= Graduate diploma/certificate;  3= Bachelor's degree;  4= Advanced diploma/diploma;  5= Certificate; 6 Other |
| Work status | Parental Labor force Status | Employment status | P1 employed by Labour Force Survey definition | 1= Employed;  2= Unemployed;  3= Not in labour force |
| Health status | General health | Global health measure | In general, how would you say child's current health is? (waves 2 to 6)  In general, how would you say your current health is? (waves 7 and 8) | 1= Excellent,  2= Very good  3= Good  4= Fair  5= Poor |

**Note: AUD= Australian dollar**

**Appendix B**

**Table 3: Association between socioeconomic (Income) inequalities and consumption of fruit and vegetables, sweet and savory foods, and drinking sugary beverages (n=3127)**

| **Variables** | **Consumption of Fruit and vegetables (Yes/No)** | | **Consumption of sweet and savory food (Yes/No** | | **Drinking sugary beverages (Yes/No)** | |
| --- | --- | --- | --- | --- | --- | --- |
|  | **Crude OR (95%CI)** | **Adj. OR (95%CI)** | **Crude OR (95%CI** | **Adj. OR (95%CI)** | **Crude OR (95%CI** | **Adj. OR (95%CI)** |
| **Socioeconomic status (Ref: low SES)** | | | | | | |
| High SES | 2.71(1.7,4.3) | 3.26(2.04,5.2) | 0.82 (0.66,1.02) | 0.9 (0.72,1.12) | 0.59 (0.51,0.69) | 0.66 (0.57,0.77) |
| **Age Groups (Ref: children)** | | | | | | |
| Adolescence | 0.55(0.49,0.62) | 0.5 (0.44,0.56) | 0.7 (0.64,0.76) | 0.7 (0.64,0.76) | 0.63 (0.59,0.67) | 0.67 (0.63,0.71) |
| **Gender (Ref: Male)** | | | | | | |
| Female | 1.08(0.96,1.22) | 1.07(0.95,1.21) | 0.96 (0.89,1.04) | 0.96 (0.88,1.04) | 0.92 (0.87,0.97) | 0.91 (0.86,0.97) |
| **Place of residence (Ref: accessible)** | | | | | | |
| Not accessible | 1.01(0.75,1.35) | 0.94(0.7,1.26) | 0.94 (0.78,1.15) | 0.91 (0.75,1.11) | 1 (0.87,1.15) | 0.97 (0.84,1.11) |
| **Ethnicity (Ref: no aboriginal)** | | | | | | |
| Aboriginal | 0.52(0.4,0.68) | 0.58(0.44,0.76) | 1.06 (0.83,1.34) | 1.02 (0.8,1.3) | 1.7 (1.41,2.04) | 1.62 (1.35,1.95) |
| **Languages spoken (Ref: English**) | | | | | | |
| Other than English | 1.51(1.22,1.88) | 1.57(1.26,1.96) | 0.91 (0.8,1.03) | 0.89 (0.79,1.01) | 1.37 (1.24,1.5) | 1.29 (1.17,1.42) |
| **Number of Siblings (Ref: One child)** | | | | | | |
| Two children | 1.12(0.98,1.28) | 1.2 (1.04,1.37) | 1.07 (0.98,1.17) | 1.07 (0.98,1.18) | 0.89 (0.83,0.94) | 0.88 (0.82,0.94) |
| Three children | 0.99(0.81,1.21) | 1.14(0.94,1.4) | 1.09 (0.94,1.25) | 1.09 (0.95,1.26) | 0.97 (0.88,1.06) | 0.91 (0.82,1.01) |
| four children | 0.92(0.68,1.25) | 1.13(0.83,1.54) | 1.18 (0.94,1.48) | 1.19 (0.94,1.5) | 0.88 (0.75,1.02) | 0.77 (0.66,0.9) |
| **General health status (Ref: Excellent)** | | | | | | |
| Poor health | 0.52 (0.38,0.72) | 0.58 (0.42,0.8) | 0.86 (0.66,1.12) | 0.89 (0.68,1.16) | 0.88(0.73,1.07) | 0.85 (0.7,1.04) |

**Table 3A: Association between socioeconomic (Education) inequalities and consumption of fruit and vegetables, sweet and savory foods, and drinking sugary beverages (n=3127)**

| **Variables** | **Consumption of Fruit and vegetables (Yes/No)** | | **Consumption of sweet and savory food (Yes/No** | | **Drinking sugary beverages (Yes/No)** | |
| --- | --- | --- | --- | --- | --- | --- |
|  | **Crude OR (95%CI)** | **Adj. OR (95%CI)** | **Crude OR (95%CI** | **Adj. OR (95%CI)** | **Crude OR (95%CI** | **Adj. OR (95%CI)** |
| **Socioeconomic status (Ref: low SES)** | | | | | | |
| High SES | **1.06(0.93,1.2)** | **1.05(0.93,1.2)** | **1.16 (1.07,1.27)** | **1.15 (1.05,1.25)** | **0.53 (0.61,0.822)** | **0.56(0.63,0.83)** |
| **Age Groups (Ref: children)** | | | | | | |
| Adolescence | 0.55(0.49,0.62) | 0.55(0.49,0.62) | 0.7 (0.64,0.76) | 0.7 (0.64,0.76) | 0.63 (0.59,0.67) | 0.64(0.6,0.68) |
| **Gender (Ref: Male)** | | | | | | |
| Female | 1.08(0.96,1.22) | 1.08(0.96,1.22) | 0.96 (0.89,1.04) | 0.96 (0.89,1.04) | 0.92 (0.87,0.97) | 0.91(0.86,0.97) |
| **Place of residence (Ref: accessible)** | | | | | | |
| Not accessible | 1.01(0.75,1.35) | 0.95(0.71,1.27) | 0.94 (0.78,1.15) | 0.91 (0.75,1.11) | 1 (0.87,1.15) | 0.96(0.83,1.11) |
| **Ethnicity (Ref: no aboriginal)** | | | | | | |
| Aboriginal | 0.52(0.4,0.68) | 0.52(0.4,0.68) | 1.057 (0.83,1.34) | 1.01(0.7, 1.28) | 1.7 (1.41,2.04) | 1.71(1.42,2.06) |
| **Languages spoken (Ref: English**) | | | | | | |
| Other than English | 1.51(1.22,1.88) | 1.46(1.17,1.82) | 0.91 (0.8,1.03) | 0.9 (0.79,1.02) | 1.37 (1.24,1.5) | 1.34(1.22,1.48) |
| **Number of Siblings (Ref: One child)** | | | | | | |
| Two children | 1.12(0.98,1.28) | 1.15(1,1.32) | 1.07 (0.98,1.17) | 1.07 (0.98,1.17) | 0.89 (0.83,0.94) | 0.89(0.84,0.95) |
| Three children | 0.99(0.81,1.21) | 1.02(0.84,1.25) | 1.09 (0.94,1.25) | 1.09 (0.94,1.25) | 0.97 (0.88,1.06) | 0.95(0.86,1.05) |
| four children | 0.92(0.68,1.25) | 0.96(0.7,1.3) | 1.18 (0.94,1.48) | 1.18 (0.94,1.49) | 0.88 (0.75,1.02) | 0.84(0.72,0.97) |
| **General health status (Ref: Excellent)** | | | | | | |
| Poor health | 0.52(0.38,0.72) | 0.54(0.4,0.75) | 0.86 (0.66,1.12) | 0.88 (0.68,1.15) | 0.81(0.73,1.09) | 0.83 (0.7,1.07) |

**Table 3B: Association between socioeconomic (Employment) inequalities and consumption of fruit and vegetables, sweet and savory foods, and drinking sugary beverages (n=3127)**

| **Variables** | **Consumption of Fruit and vegetables (Yes/No)** | | **Consumption of sweet and savory food (Yes/No** | | **Drinking sugary beverages (Yes/No)** | |
| --- | --- | --- | --- | --- | --- | --- |
|  | **Crude OR (95%CI)** | **Adj. OR (95%CI)** | **Crude OR (95%CI** | **Adj. OR (95%CI)** | **Crude OR (95%CI** | **Adj. OR (95%CI)** |
| **Socioeconomic status (Ref: low SES)** | | | | | | |
| High SES | **0.87**  **(0.76,0.99)** | **0.77**  **(0.68,1.01)** | **1.09 (0.99,1.2)** | **1.02**  **(0.93,1.13)** | **1.25 (1.17,1.33)** | **1.15**  **(1.08,1.23)** |
| **Age Groups (Ref: children)** | | | | | | |
| Adolescence | 0.55  (0.49,0.62) | 0.53  (0.47,0.6) | 0.7 (0.64,0.76) | 0.7 (  0.64,0.76) | 0.63 (0.59,0.67) | 0.65  (0.61,0.69) |
| **Gender (Ref: Male)** | | | | | | |
| Female | 1.08  (0.96,1.22) | 1.08  (0.96,1.22) | 0.96 (0.89,1.04) | 0.96  (0.89,1.04) | 0.92 (0.87,0.97) | 0.91  (0.86,0.97) |
| **Place of residence (Ref: accessible)** | | | | | | |
| Not accessible | 1.01  (0.75,1.35) | 0.95  (0.71,1.27) | 0.94 (0.78,1.15) | 0.91  (0.75,1.11) | 1 (0.87,1.15) | 0.96  (0.84,1.11) |
| **Ethnicity (Ref: no aboriginal)** | | | | | | |
| Aboriginal | 0.52  (0.4,0.68) | 0.54  (0.41,0.71) | 1.05 (0.83,1.34) | 1.008  7 (0.79,1.34) | 1.7 (1.41,2.04) | 1.67  (1.39,2.01) |
| **Languages spoken (Ref: English**) | | | | | | |
| Other than English | 1.51  (1.22,1.88) | 1.5 (  1.2,1.87) | 0.91 (0.8,1.03) | 0.88  (0.78,1) | 1.37 (1.24,1.5) | 1.31  (1.19,1.44) |
| **Number of Siblings (Ref: One child)** | | | | | | |
| Two children | 1.12(0.98,1.28) | 1.17(1.02,1.34) | 1.07 (0.98,1.17) | 1.07(0.98,1.18) | 0.89 (0.83,0.94) | 0.89(0.83,0.95) |
| Three children | 0.99(0.81,1.21) | 1.06(0.87,1.3) | 1.09 (0.94,1.25) | 1.09(0.95,1.26) | 0.97 (0.88,1.06) | 0.94(0.85,1.03) |
| four children | 0.92(0.68,1.25) | 1.04(0.76,1.42) | 1.18 (0.94,1.48) | 1.17(0.93,1.48) | 0.88 (0.75,1.02) | 0.8 (0.68,0.93) |
| **General health status (Ref: Excellent)** | | | | | | |
| Poor health | 0.52(0.38,0.72) | 0.56(0.41,0.77) | 0.86 (0.66,1.12) | 0.88(0.68,1.15) | 0.88 (0.73,1.07) | 0.87(0.72,1.06) |

**Table 4: Association between socioeconomic (Income) inequalities and Leisure -time activities, outdoor activity, and sleeping problems** **(n=3127)**

| **Variables** | **Leisure-time activities (Screentime/ Active time)** | | **Outdoor activity (Yes/No)** | | **Sleep problem (Yes/No)** | |
| --- | --- | --- | --- | --- | --- | --- |
|  | **Crude OR(95%CI)** | **Adj. OR (95%CI)** | **Crude OR (95%CI)** | **Adj. OR (95%CI)** | **Crude OR (95%CI)** | **Adj. OR (95%CI)** |
| **Socioeconomic status (Ref: low SES)** | | | | | | |
| High SES | 0.65 (0.54,0.78) | 0.79(0.65,0.95) | 1.52(1.05,2.21) | 2.05(1.4,2.98) | 0.9 (0.76,1.06) | 0.88(0.74,1.04) |
| **Age Groups (Ref: children)** | | | | | | |
| Adolescence | 0.41 (0.38,0.45) | 0.42(0.39,0.46) | 0.32(0.28,0.36) | 0.29(0.25,0.32) | 0.97 (0.91,1.03) | 0.98(0.91,1.04) |
| **Gender (Ref: Male)** | | | | | | |
| Female | 1.01 (0.94,1.06) | 1.07 (0.94,1.06) | 1.07(0.95,1.21) | 1.05(0.93,1.19) | 0.97 (0.92,1.03) | 0.97(0.91,1.03) |
| **Place of residence (Ref: accessible)** | | | | | | |
| Not accessible | 0.98 (0.84,1.13) | 0.92(0.79,1.07) | 1.19(0.86,1.64) | 1.04(0.75,1.44) | 0.93 (0.8,1.07) | 0.93(0.8,1.07) |
| **Ethnicity (Ref: no aboriginal)** | | | | | | |
| Aboriginal | 1.41 (1.2,1.66) | 1.29(1.09,1.52) | 0.61(0.45,0.82) | 0.65(0.48,0.88) | 1.31 (1.12,1.54) | 1.3(1.1,1.53) |
| **Languages spoken (Ref: English**) | | | | | | |
| Other than English | 0.98 (0.89,1.08) | 0.92(0.84,1.02) | 0.95(0.78,1.15) | 0.96(0.79,1.17) | 0.95 (0.87,1.04) | 0.92(0.84,1.02) |
| **Number of Siblings (Ref: One child)** | | | | | | |
| Two children | 1.08 (1.01,1.15) | 1.08(1.01,1.15) | 1.13(0.98,1.3) | 1.21(1.05,1.4) | 0.88 (0.83,0.94) | 0.88(0.82,0.94) |
| Three children | 1.1 (0.99,1.21) | 1.08(0.97,1.19) | 0.89(0.73,1.09) | 1.05(0.85,1.29) | 0.77 (0.7,0.86) | 0.75(0.68,0.83) |
| four children | 1.29 (1.1,1.51) | 1.24(1.05,1.45) | 0.76(0.57,1.03) | 0.96(0.71,1.31) | 0.75 (0.64,0.89) | 0.71(0.6,0.84) |
| **General health status (Ref: Excellent)** | | | | | | |
| Poor health | 1.08 (0.88,1.33) | 1.12(0.91,1.37) | 0.57(0.41,0.8) | 0.67(0.47,0.94) | 3.3 (2.74,3.98) | 3.21(2.66,3.88) |

**Table 4A: Association between socioeconomic (Education) inequalities and Leisure -time activities, outdoor activity, and sleeping problems** **(n=3127)**

| **Variables** | **Leisure-time activities (Screentime/ Active time)** | | **Outdoor activity (Yes/No)** | | **Sleep problem (Yes/No)** | |
| --- | --- | --- | --- | --- | --- | --- |
|  | **Crude OR(95%CI)** | **Adj. OR (95%CI)** | **Crude OR (95%CI)** | **Adj. OR (95%CI)** | **Crude OR (95%CI)** | **Adj. OR (95%CI)** |
| **Socioeconomic status (Ref: low SES)** | | | | | | |
| High SES | **1.17 (1.01,1.25)** | **1.18(1.01,1.26)** | **0.83(0.73,0.98)** | **0.8 (0.7,0.99)** | **1.04 (0.98,1.11)** | **1.03(0.97,1.09)** |
| **Age Groups (Ref: children)** | | | | | | |
| Adolescence | 0.41 (0.38,0.45) | 0.41(0.38,0.44) | 0.32(0.28,0.36) | 0.31  (0.28,0.35) | 0.97 (0.91,1.03) | 0.96(0.9,1.02) |
| **Gender (Ref: Male)** | | | | | | |
| Female | 1.01 (0.94,1.069) | 1.01 (0.94,1.06) | 1.07(0.95,1.21) | 1.07(0.94,1.21) | 0.97 (0.92,1.03) | 0.97(0.91,1.03) |
| **Place of residence (Ref: accessible)** | | | | | | |
| Not accessible | 0.98 (0.84,1.13) | 0.92(0.79,1.07) | 1.19(0.86,1.64) | 1.06(0.76,1.47) | 0.93 (0.8,1.07) | 0.92(0.8,1.07) |
| **Ethnicity (Ref: no aboriginal)** | | | | | | |
| Aboriginal | 1.41 (1.2,1.66) | 1.28(1.08,1.51) | 0.61(0.45,0.82) | 0.58(0.43,0.78) | 1.31 (1.12,1.54) | 1.33(1.13,1.56) |
| **Languages spoken (Ref: English**) | | | | | | |
| Other than English | 0.98 (0.89,1.08) | 0.94(0.85,1.04) | 0.95(0.78,1.15) | 0.86(0.71,1.05) | 0.95 (0.87,1.04) | 0.94(0.86,1.04) |
| **Number of Siblings (Ref: One child)** | | | | | | |
| Two children | 1.08 (1.01,1.15) | 1.09(1.01,1.16) | 1.13(0.98,1.3) | 1.16(1.01,1.34) | 0.88 (0.83,0.94) | 0.88(0.83,0.94) |
| Three children | 1.1 (0.99,1.21) | 1.1 (0.99,1.22) | 0.89  (0.73,1.09) | 0.94(0.77,1.15) | 0.77 (0.7,0.86) | 0.77(0.69,0.85) |
| four children | 1.29 (1.1,1.51) | 1.26(1.08,1.48) | 0.76(0.57,1.03) | 0.8 (0.59,1.08) | 0.75 (0.64,0.89) | 0.74(0.63,0.87) |
| **General health status (Ref: Excellent)** | | | | | | |
| Poor health | 1.08 (0.88,1.33) | 1.11(0.9,1.37) | 0.57(0.41,0.8) | 0.61(0.44,0.87) | 3.3 (2.74,3.98) | 3.27(2.71,3.94) |

**Table 4B: Association between socioeconomic (Employment) inequalities and Leisure -time activities, outdoor activity, and sleeping problems** **(n=3127)**

| **Variables** | **Leisure-time activities (Screentime/ Active time)** | | **Outdoor activity (Yes/No)** | | **Sleep problem (Yes/No)** | |
| --- | --- | --- | --- | --- | --- | --- |
|  | **Crude OR(95%CI)** | **Adj. OR (95%CI)** | **Crude OR (95%CI)** | **Adj. OR (95%CI)** | **Crude OR (95%CI)** | **Adj. OR (95%CI)** |
| **Socioeconomic status (Ref: low SES)** | | | | | | |
| High SES | 1.21 (0.93,1.29) | 1.05(0.98,1.32) | 0.85(0.74,0.97) | 0.69(0.6,0.8) | 1.03 (1.06,1.21) | 1.10  (1.08,1.24) |
| **Age Groups (Ref: children)** | | | | | | |
| Adolescence | 0.41 (0.38,0.45) | 0.41(0.38,0.45) | 0.32  (0.28,0.36) | 0.3 (  0.26,0.34) | 0.97 (0.91,1.03) | 0.98  (0.92,1.05) |
| **Gender (Ref: Male)** | | | | | | |
| Female | 1.01 (0.94,1.06) | 1.01  2 (0.94,1.06) | 1.07  (0.95,1.21) | 1.07  (0.94,1.21) | 0.97 (0.92,1.03) | 0.97  (0.91,1.02) |
| **Place of residence (Ref: accessible)** | | | | | | |
| Not accessible | 0.98 (0.84,1.13) | 0.92(0.79,1.07) | 1.19(0.86,1.64) | 1.05(0.76,1.46) | 0.93 (0.8,1.07) | 0.92(0.8,1.07) |
| **Ethnicity (Ref: no aboriginal)** | | | | | | |
| Aboriginal | 1.41 (1.2,1.66) | 1.31(1.11,1.55) | 0.61(0.45,0.82) | 0.61(0.45,0.82) | 1.31 (1.12,1.54) | 1.3 (1.1,1.53) |
| **Languages spoken (Ref: English**) | | | | | | |
| Other than English | 0.98 (0.89,1.08) | 0.93(0.84,1.02) | 0.95(0.78,1.15) | 0.91(0.75,1.11) | 0.95 (0.87,1.04) | 0.93(0.84,1.02) |
| **Number of Siblings (Ref: One child)** | | | | | | |
| Two children | 1.08 (1.01,1.15) | 1.08(1.01,1.16) | 1.13(0.98,1.3) | 1.18(1.02,1.37) | 0.88 (0.83,0.94) | 0.87(0.82,0.93) |
| Three children | 1.1 (0.99,1.21) | 1.1 (0.99,1.22) | 0.89(0.73,1.09) | 0.98(0.8,1.2) | 0.77 (0.7,0.86) | 0.75(0.68,0.83) |
| four children | 1.29 (1.1,1.51) | 1.26(1.07,1.48) | 0.76(0.57,1.03) | 0.9 (0.66,1.22) | 0.75 (0.64,0.89) | 0.71(0.6,0.84) |
| **General health status (Ref: Excellent)** | | | | | | |
| Poor health | 1.08 (0.88,1.33) | 1.13(0.91,1.39) | 0.57(0.41,0.8) | 0.64(0.45,0.9) | 3.3 (2.74,3.98) | 3.23(2.68,3.9) |

**Table 5: Association between socioeconomic (Income) inequalities and smoking tobacco, and drinking alcohol in adolescents(n=3127)**

| **Variables** | **smoking tobacco (Yes/No)** | | **Drinking alcohol (Yes/No)** | |
| --- | --- | --- | --- | --- |
|  | **Crude OR (95%CI)** | **Adj. OR (95%CI)** | **Crude OR (95%CI)** | **Adj. OR (95%CI)** |
| **Socioeconomic status (Ref: low SES)** | | | | |
| High SES | 0.59(0.32,1.09) | 0.6 (0.33,1.11) | 1.07(0.84,1.36) | 1.02(0.8,1.31) |
| **Gender (Ref: Male)** | | | | |
| Female | 0.96(0.76,1.21) | 0.97 0.77,1.22) | 1.07(0.97,1.19) | 1.08(0.97,1.19) |
| **Place of residence (Ref: accessible)** | | | | |
| Not accessible | 0.86(0.42,1.77) | 0.83 0.41,1.71) | 0.77(0.56,1.06) | 0.76(0.55,1.04) |
| **Ethnicity (Ref: no aboriginal)** | | | | |
| Aboriginal | 2.87(1.75,4.71) | 2.65 (1.6,4.37) | 0.92(0.65,1.28) | 0.91(0.65,1.28) |
| **Languages spoken (Ref: English**) | | | | |
| Other than English | 0.54(0.32,0.91) | 0.5 (0.29,0.85) | 0.68(0.56,0.83) | 0.67(0.55,0.82) |
| **Number of Siblings (Ref: One child)** | | | | |
| Two children | 0.99(0.76,1.28) | 0.97 0.74,1.25) | 0.92(0.82,1.04) | 0.91(0.81,1.02) |
| Three children | 0.95(0.64,1.42) | 0.89 (0.6,1.33) | 0.89(0.74,1.06) | 0.89(0.74,1.07) |
| four children | 1.03(0.55,1.93) | 0.91 0.48,1.72) | 0.9(0.68,1.2) | 0.91(0.68,1.21) |
| **General health status (Ref: Excellent)** | | | | |
| Poor health | 2.67(1.61,4.43) | 2.6 (1.56,4.33) | 1.11(0.8,1.54) | 1.12(0.8,1.55) |

**Table 5A: Association between socioeconomic (Education) inequalities and smoking tobacco, and drinking alcohol in adolescents(n=3127)**

| **Variables** | **smoking tobacco (Yes/No)** | | **Drinking alcohol (Yes/No)** | |
| --- | --- | --- | --- | --- |
|  | **Crude OR (95%CI)** | **Adj. OR (95%CI)** | **Crude OR (95%CI)** | **Adj. OR (95%CI)** |
| **Socioeconomic status (Ref: low SES)** | | | | |
| High SES | 1.31 (1.03,1.66) | 1.24(0.97,1.57) | 1.04(0.93,1.16) | 1.04(0.93,1.16) |
| **Gender (Ref: Male)** | | | | |
| Female | 0.96 (0.76,1.21) | 0.96(0.76,1.21) | 1.07(0.97,1.19) | 1.08(0.97,1.19) |
| **Place of residence (Ref: accessible)** | | | | |
| Not accessible | 0.86 (0.42,1.77) | 0.82(0.4,1.69) | 0.77(0.56,1.06) | 0.76(0.55,1.04) |
| **Ethnicity (Ref: no aboriginal)** | | | | |
| Aboriginal | 2.87 (1.75,4.71) | 2.7(1.63,4.45) | 0.92(0.65,1.28) | 0.91(0.65,1.28) |
| **Languages spoken (Ref: English**) | | | | |
| Other than English | 0.54 (0.32,0.91) | 0.53  (0.31,0.9) | 0.68  (0.56,0.83) | 0.68  (0.56,0.82) |
| **Number of Siblings (Ref: One child)** | | | | |
| Two children | 0.99 (0.76,1.28) | 0.99  (0.76,1.28) | 0.92  (0.82,1.04) | 0.91  (0.81,1.03) |
| Three children | 0.95 (0.64,1.42) | 0.94(0.63,1.41) | 0.89(0.74,1.06) | 0.9 (0.75,1.07) |
| four children | 1.03 (0.55,1.92) | 0.9988 (0.53,1.87) | 0.9 (0.68,1.2) | 0.92(0.69,1.23) |
| **General health status (Ref: Excellent)** | | | | |
| Poor health | 2.67 (1.61,4.43) | 2.66(1.6,4.43) | 1.11(0.8,1.54) | 1.12(0.81,1.56) |

**Table 5B: Association between socioeconomic (Employment) inequalities and smoking tobacco, and drinking alcohol in adolescents(n=3127)**

| **Variables** | **smoking tobacco (Yes/No)** | | **Drinking alcohol (Yes/No)** | |
| --- | --- | --- | --- | --- |
|  | **Crude OR (95%CI)** | **Adj. OR (95%CI)** | **Crude OR (95%CI)** | **Adj. OR (95%CI)** |
| **Socioeconomic status (Ref: low SES)** | | | | |
| High SES | 1.24(0.92,1.67) | 1.21(0.89,1.64) | 0.86(0.75,1) | 0.89(0.77,1.03) |
| **Gender (Ref: Male)** | | | | |
| Female | 0.96(0.76,1.21) | 0.95(0.76,1.2) | 1.07(0.97,1.19) | 1.08(0.97,1.19) |
| **Place of residence (Ref: accessible)** | | | | |
| Not accessible | 0.86(0.42,1.77) | 0.83(0.4,1.7) | 0.77(0.56,1.06) | 0.76(0.55,1.04) |
| **Ethnicity (Ref: no aboriginal)** | | | | |
| Aboriginal | 2.87(1.75,4.71) | 2.76(1.68,4.56) | 0.92(0.65,1.28) | 0.94(0.67,1.31) |
| **Languages spoken (Ref: English**) | | | | |
| Other than English | 0.54(0.32,0.91) | 0.52(0.31,0.88) | 0.68(0.56,0.83) | 0.68(0.56,0.83) |
| **Number of Siblings (Ref: One child)** | | | | |
| Two children | 0.99(0.76,1.28) | 0.98(0.75,1.27) | 0.92(0.82,1.04) | 0.92(0.82,1.03) |
| Three children | 0.95(0.64,1.42) | 0.93(0.62,1.38) | 0.89(0.74,1.06) | 0.91(0.76,1.09) |
| four children | 1.03(0.55,1.93) | 0.96(0.51,1.81) | 0.9(0.68,1.2) | 0.95(0.71,1.27) |
| **General health status (Ref: Excellent)** | | | | |
| Poor health | 2.67(1.61,4.43) | 2.66(1.6,4.43) | 1.11(0.8,1.54) | 1.14(0.82,1.58) |
